# Clinical procedures and practices used in the perioperative treatment of female genital fistula during a prospective cohort study

**DOI:** 10.1186/1471-2393-14-220

**Published:** 2014-07-05

**Authors:** Joseph K Ruminjo, Veronica Frajzyngier, Muhammad Bashir Abdullahi, Frank Asiimwe, Thierno Hamidou Barry, Abubakar Bello, Dantani Danladi, Sanda Oumarou Ganda, Sa’ad Idris, Maman Inoussa, Maura Lynch, Felicity Mussell, Dulal Chandra Podder, Abba Wali, Mark A Barone

**Affiliations:** 1 EngenderHealth, 440 9th Avenue, FL 13, New York, NY 10001, USA; 2 Faridat Yakubu Hospital, Gusau, Zamfara State, Nigeria; 3 Kagando Hospital, Kagando, Kasese District, Uganda; 4 Prefectoral Hospital of Kissidougou, Kissidougou, Guinea; 5 Maryam Abacha Hospital, Sokoto, Sokoto State, Nigeria; 6 Specialist Fistula Centre Birnin Kebbi, Birnin Kebbi, Kebbi State, Nigeria; 7 Lamorde Hospital, Niamey, Niger; 8 Maradi Hospital, Maradi, Niger; 9 Kitovu Mission Hospital, Kampala, Masaka, Uganda; 10 LAMB Hospital, Rangpur Division, Parbatipur, Bangladesh; 11 Kumudini Hospital, Narayanganj, Bangladesh

**Keywords:** Female genital fistula, Fistula repair, Clinical practices, Perioperative procedures, Fistula treatment

## Abstract

**Background:**

Treatment and care for female genital fistula have become increasingly available over the last decade in countries across Africa and South Asia. Before the International Federation of Gynaecology and Obstetrics (FIGO) and partners published a global fistula training manual in 2011 there was no internationally recognized, standardized training curriculum, including perioperative care. The community of fistula care practitioners and advocates lacks data about the prevalence of various perioperative clinical procedures and practices and their potential programmatic implications are lacking.

**Methods:**

Data presented here are from a prospective cohort study conducted between September 2007 and September 2010 at 11 fistula repair facilities supported by Fistula Care in five countries. Clinical procedures and practices used in the routine perioperative management of over 1300 women are described.

**Results:**

More than two dozen clinical procedures and practices were tabulated. Some of them were commonly used at all sites (e.g., vaginal route of repair, 95.3% of cases); others were rare (e.g., flaps/grafts, 3.4%) or varied widely depending on site (e.g. for women with urinary fistula, the inter-quartile range for median duration of post-repair bladder catheterization was 14 to 29 days).

**Conclusions:**

These findings show a wide range of clinical procedures and practices with different program implications for safety, efficacy, and cost-effectiveness. The variability indicates the need for further research so as to strengthen the evidence base for fistula treatment in developing countries.

## Background

In resource poor settings, obstructed labor is one of the most common causes of maternal death [1,2]. Among women who survive obstructed labor, many suffer severe and long-term sequelae; genital fistula is one of the most devastating. The global magnitude of the problem is not accurately known. The commonly cited estimate of over 2 million women living with fistula, and 50,000–100,000 new cases yearly seem to go back to a 1993 paper by Waaldijk and Armiy’u [3]; but varying more recent figures have been cited, including Dolea and AbouZahr’s model-based global estimate of 0.8% of women aged 15–44 years or 2.15% of what they described as “neglected” obstructed labor cases – the predominant cause of fistula - and global estimates of women living with fistula ranging from 654,000 to 3.5 million [4]–[6].

Before the International Federation of Gynaecology and Obstetrics (FIGO) and partners published a global fistula training manual in 2011 [5] there was no internationally recognized, standardized training curriculum. In general, fistula surgeons and their teams have developed their own clinical procedures and practices for repair of genital fistula, drawing upon personal experience and trying to relate them to basic surgical principles. Various textbooks, specialist practical and training manuals, and the published literature - including a report by an international consultation on vesicovaginal fistula sponsored by the Societe Internationale d’Urologie and the International Consultation on Urological Diseases (SIU-ICUD) [6] - describe a range of procedures and practices. Consequently, there are a wide variety of pre-, intra- and postoperative procedures and practices in use today at fistula service sites. Indeed, Arrowsmith et al. surveyed fistula surgeons working in resource-poor settings about clinical practices and found wide variations in perioperative interventions; use of prophylactic antibiotics and postoperative urinary bladder catheterization were the only consistently reported practices [7]. Aside from such self-reported data by fistula surgeons, the current literature includes little evidence about what actually happens during the management of female genital fistula. The present paper tries to fill this gap.

We conducted a descriptive analysis of data on clinical procedures and practices gathered during a prospective cohort study about determinants of postoperative outcomes of female genital fistula repair surgery. The goals of this analysis were to describe procedures and practices in different contexts facilitating a better understanding of clinical realities, to compare the findings to what is described in the literature, and to identify potential areas for further research.

## Methods

Data presented here are from a prospective cohort study designed to identify predictors of female genital fistula repair outcomes. The study was conducted among women seeking fistula repair services between September 2007 and September 2010 at 11 sites in five countries: Bangladesh (three sites), Guinea (one site), Niger (two sites), Nigeria (three sites), and Uganda (two sites). Site selection was purposive, and comprised fistula repair facilities supported by Fistula Care, an EngenderHealth project funded by the United States Agency for International Development.

Details of the study methods are presented elsewhere [8]. Briefly, the study included women who had a urinary fistula, a fecal fistula (all of them rectovaginal, RVF), or both. Fistulae of obstetric or traumatic origin were included. Study sites used their standard procedures and practices before, during, and after repair surgery. Data were collected during clinical examinations and interviews and reported on standardized forms at admission to hospital, during the hospital stay, at discharge, and at the three-month follow-up visit. Three month follow-up was routine practice at eight of the 11 study sites. However, we asked all sites to conduct a three month follow-up visit as part of the study.

**Figure 1:**
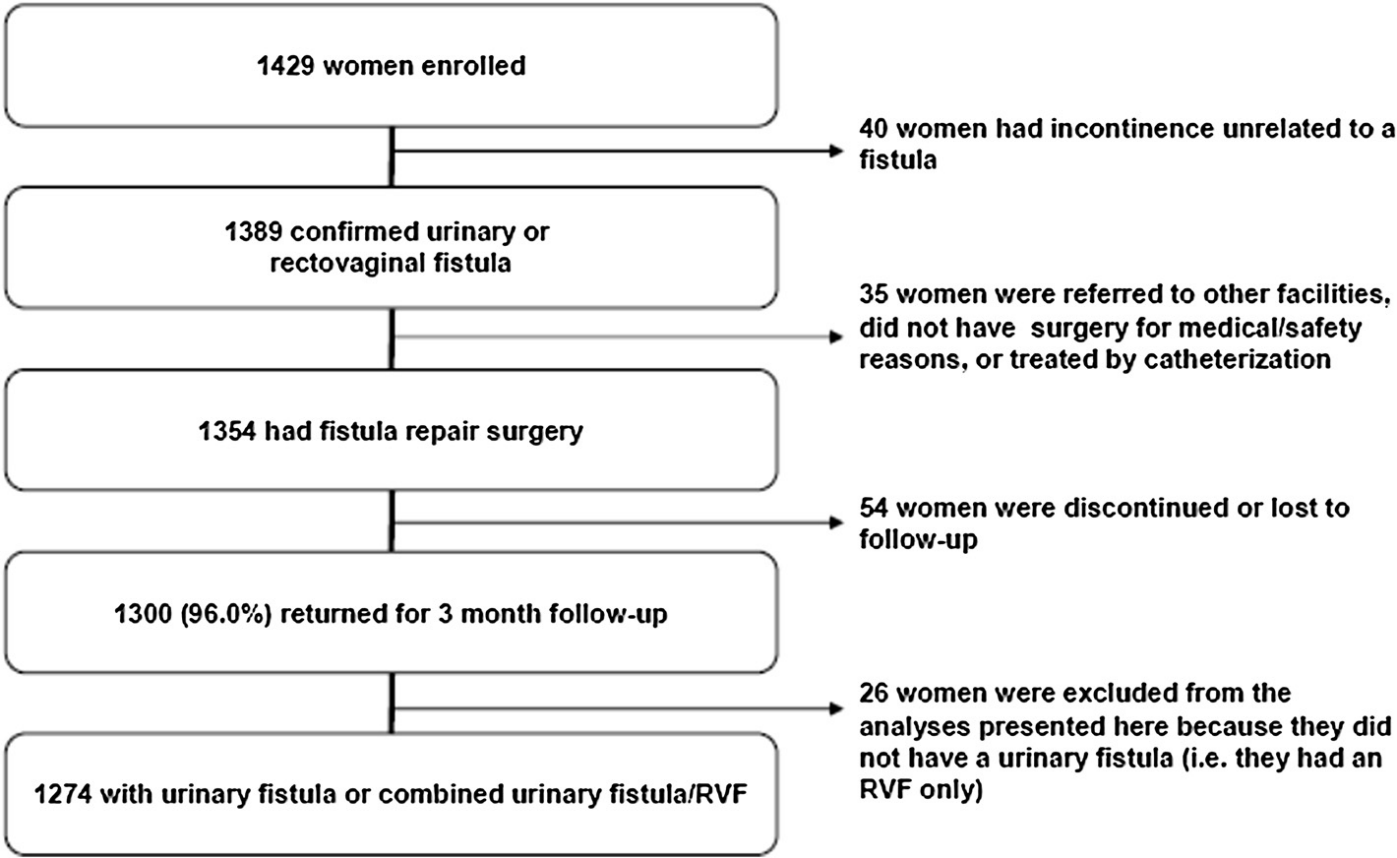
**Study participants’ flow chart.** Data presented in this paper are from a prospective cohort study designed to identify predictors of female genital fistula repair outcomes. This figure details the flow of participants in the study. The 1429 women were enrolled at 11 sites in Bangladesh, Guinea, Niger, Nigeria and Uganda.

The study protocol was reviewed and approved as required by institutional and government guidelines in the countries where study sites were located. Approvals were obtained from the Comite National d’Ethique pour la Recherche en Sante in Guinea; la Ministere de la Sante Publique in Niger; the National Health Research Ethics Committee in Nigeria; and the National Council for Science and Technology in Uganda. The Bangladesh Medical Research Council declined submission to review the study on the basis of its observational nature.Figure 1 shows the flow of study participants. A total of 1429 women were enrolled, ranging from 51 to 261 women per site at 10 of the sites, the variability based primarily on the site’s caseload. The eleventh site enrolled only five women before fistula repair services were suspended because the sole fistula surgeon departed (data for these women were included in the analyses presented here).

Data presented here are from the 1354 women who underwent fistula repair surgery during the study; the number of cases for specific procedures and practices varied slightly because of missing data for some variables. Women with RVF and no urinary fistula were excluded from analyses that were pertinent only to women with urinary fistula; e.g. drinking regimens prescribed, use of ureteric catheters, ureteric reimplantation, use of flaps/grafts, bladder sutures, duration of bladder catheterization, bladder training and dye test at the end of surgery, at catheter removal/discharge, and at 3 month follow-up). Descriptive data are presented by study site solely to show range in practice; tests of statistical significance are not used.

## Results and discussion

### Sociodemographic data and fistula characteristics

Detailed sociodemographic data of study participants and the characteristics of their fistula are reported elsewhere [8,9]. A brief summary is provided here for context. Median age for women at time of repair was 25 years (IQR 20–35). At the time of fistula repair, over half (887/1334, 66.5%) of women reported they were married; 26.7% (356/1334) reported they were divorced or separated, most after fistula occurred. Median age at first marriage was 15.0 years (IQR 14.0–18.0). Most women (1149/1339, 85.8%) were from rural areas; approximately one-fifth (279/1351, 20.7%) had at least primary education. Median parity was 2.0 (IQR 1.0–5.0). Approximately one-fourth (22.9%, 310/1351) of women reported having a previous repair attempt, with a median of 1.0 (IQR 1.0–2.0) attempts. Almost all women (1311/1352, 97.0%) reported the fistula occurred after delivery.

Over 90% of women (1290/1354, 95.3%) had a urinary fistula; 3.1% (42/1354) had combined urinary and rectovaginal fistulae, and 1.6% (22/1354) had RVF without a urinary fistula. While few women (99/1319, 7.5%) had severe vaginal scarring, nearly 30% (349/1218, 28.7%) had a small bladder, and 40% (529/1316) had some involvement of the urethra. Only 5.8% (77/1318) of women had more than one urinary fistula.

Nearly 70% of urinary fistulae (895/1308, 68.4%) were midvaginal, juxta-urethral, or juxta-cervical. According to the surgeons’ subjective assessment during exam under anesthesia, similar numbers of fistulae were categorized as simple (372/1312, 28.4%) or complex (376/1312, 28.7%). The remaining 43.0% (564/1312) were classified as intermediate.

### Preoperative procedures and practices

#### Examination under anesthesia (EUA)

EUA was performed as part of and just before fistula surgery for 96.9% (1298/1340) of women. It was a separate independent procedure for only 3.1% (42/1340) of women, either because that was the site’s routine practice (34.1%), fistula was hard to access (31.8%), pelvic examination was too painful (13.6%), severe vaginal scarring was present (9.1%), fistula was too small (4.5%), or, conversely, too large (4.5%). Waaldijk strongly discourages routine performance of separate preoperative EUA because of the extra requirements for time, anesthesia, expenses and delay of definitive treatment for the fistula [10]. However, surgeons, especially those not completely confident in being able to manage unexpected findings during repair surgery, may feel more comfortable if they perform a separate EUA so as to better prepare and, if necessary, refer to a more experienced practitioner.

#### Preoperative enema

Preoperative enema was administered to 24.9% of women (322/1295). For women with urinary fistula, the proportion was 24.3% (300/1234); for RVF, it was 38.1% (8/21); and for combined fistula, it was 35.0% (14/40). Ideally, the rectum should be empty before surgery to ensure a clean operating field and reduce chances of contamination; the FIGO Manual states enema as optional for urinary fistula but essential for RVF [5]. In our study, however, an enema was not routinely performed. Hancock also found that enema is not routine and is sometimes given at the last minute, leading to soiling during surgery and necessitating a temporary anal purse-string suture [11]. He proposed the option, if enema is not given the night before, that the woman be asked to empty her bowels before coming to theater.

### Intraoperative procedures and practices

#### Type of anesthesia

Almost all repairs were conducted under spinal anesthesia (1304/1342, 97.2%), the standard for most fistula repair surgery [5,6]. Only 30 (2.2%) were done under general anesthesia (GA); eight (0.6%) used combined methods. We did not ask surgeons to detail reasons for choosing one method over another.

Spinal anesthesia generally does not require elaborate equipment; is easy to learn, relatively safe, and inexpensive; and can be administered by the surgeon. Waaldijk reported having performed more than 5000 procedures under spinal anesthesia over a 10-year period with few complications [10]. General anesthesia can be complicated, expensive, and unsafe for low-resource facilities that may not have adequate equipment, supplies, and trained anesthetists. However it is advantageous to have general anesthesia and supportive resources available because select cases may need them.

#### Surgical route of repair

Most fistulae can be closed through the vaginal route, and almost all repairs used a vaginal approach (1278/1341, 95.3%) (Table 1); few used an abdominal (52/1341, 3.9%) or combined approach (11/1341, 0.8%). All abdominal repairs were in women who had a urinary fistula and no RVF.

**Table 1:** Select perioperative procedures and practices, by site (all women in the study, unless otherwise noted)

	**Site N (%)**											
**Characteristic**	**Total (n = 1354)**	**BG 1 (n = 85)**	**BG 2 (n = 50)**	**BG 3 (n = 5)**	**GU (n = 251)**	**NI 1 (n = 74)**	**NI 2 (n = 97)**	**NG 1 (n = 59)**	**NG 2 (n = 210)**	**NG 3 (n = 153)**	**UG 1 (n = 165)**	**UG 2 (n = 205)**
Preoperative procedures												
Drinking regimen prescribed (Urinary fistula only; n = 1314)	1054 (80.2)	33 (39.3)	48 (100.0)	0 (00.0)	245 (99.6)	67 (100.0)	0 (00.0)	29 (51.8)	138 (67.0)	151 (100.0)	156 (98.1)	187 (92.1)
Median liters of fluid/day prescribed (IQR) (Urinary fistula only) n = 1059)	5 (4–8)	5 (5–5)	4 (4–4)	N/A	4 (3–5)	6 (6–6)	N/A	8 (6–8)	10 (7–10)	8 (8–8)	3 (3–3)	5 (4–5)
Prophylactic antibiotics provided (n = 1340)	552 (41.2)	48 (57.1)	50 (100.0)	3 (60.0)	251 (100.0)	2 (2.9)	48 (51.6)	4 (7.0)	9 (4.3)	34 (22.37)	28 (17.0)	75 (36.6)
Intraoperative procedures												
Vaginal route (n = 1341)	1278 (95.3)	72 (85.7)	48 (96.0)	5 (100.0)	243 (96.8)	69 (100.0)	92 (98.9)	55 (96.5)	209 (99.5)	153 (100.0)	138 (83.6)	194 (95.1)
Relaxing incision(s) used (n = 1261)	16 (1.3)	0 (00.0)	2 (4.3)	1 (20.0)	0 (00.0)	0 (00.0)	0 (00.0)	3 (5.5)	6 (3.0)	1 (0.7)	1 (00.7)	3 (1.6)
Bladder suture in single layer bladder (Urinary fistula only; n = 1271)	820 (64.5)	65 (80.3)	28 (59.6)	5 (100.0)	30 (12.2)	65 (97.0)	26 (32.9)	49 (89.1)	169 (84.1)	92 (61.3)	118 (80.8)	173 (89.2)
Bladder suture in double layers (Urinary fistula only; n = 1265)	428 (33.8)	15 (19.0)	19 (40.4)	0 (00.0)	215 (87.4)	1 (1.5)	53 (66.3)	3 (5.6)	17 (8.5)	58 (38.7)	24 (16.6)	23 (11.9)
Vaginal wall suture in single layer (Urinary fistula only; n = 1269)	1076 (84.8)	73 (90.1)	47 (100.0)	5 (100.0)	244 (99.2)	66 (98.5)	29 (36.7)	44 (80.0)	174 (87.4)	132 (88.0)	120 (82.2)	142 (73.2)
Vaginal wall suture in double layers (Urinary fistula only; n = 1261)	14 (1.1)	1 (1.3)	0 (00.0)	0 (00.0)	1 (0.4)	0 (00.0)	2 (2.6)	1 (1.9)	1 (0.5)	0 (00.0)	3 (2.1)	5 (2.6)
Flaps and grafts used (only Martius) (Urinary fistula only; n = 1263)	43 (3.4)	4 (5.1)	6 (12.8)	0 (00.0)	17 (6.9)	0 (00.0)	0 (00.0)	0 (00.0)	15 (7.5)	0 (00.0)	0 (00.0)	1 (0.5)
Prophylactic antibiotics provided (n = 1340)	843 (62.9)	59 (69.4)	5 (10.0)	5 (100.0)	251 (100.0)	66 (95.7)	87 (94.6)	0 (0.00)	4 (1.9)	14 (9.2)	160 (97.0)	192 (94.1)
Postoperative procedures												
Median duration bed-rest prescribed (n = 1331)	1 (1–3)	2 (2–2)	0 (0–0)	0 (0–8)	5 (3–7)	1 (1–1)	3 (3–13)	1 (1–1)	1 (0–1)	1 (1–1)	1 (1–1)	3 (2–3)
Drinking regimen prescribed (Urinary fistula only; n = 1304)	1148 (88.0)	60 (74.1)	48 (100.0)	0 (00. 0)	246 (100.0)	67 (100. 0)	0 (00.0)	7 (13.0)	208 (100.0)	150 (100.0)	160 (100.0)	202 (100.0)
Median liters of fluid/day prescribed (Urinary fistula only n = 1138)	5 (4–8)	4 (3–5)	4 (4–4)	N/A	3 (3–3)	10 (10–10)	N/A	8 (6–10)	10 (10–10)	8 (8–8)	6 (6–6)	5 (5–5)
Open drainage system (Urinary fistula only; n = 1310)	899 (68.6)	0 (00.0)	0 (00.0)	1 (20.0)	95 (38.6)	67 (100.0)	0 (00.0)	41 (74.5)	203 (100. 0)	151 (100. 0)	147 (91.9)	194 (95.6)
Median days of catheterization (IQR) (Urinary fistula only; n = 1294)	21.0 (14–27)	21.0 (17–21)	14.0 (14–20)	14.0 (14–14)	15.0 (14–21)	28.0 (28–28)	14.0 (13–15)	29.0 (28–34)	23.0 (21–28)	28.0 (28–28)	14.0 (14–21)	15.0 (14–16)
Bladder training performed (Urinary fistula only; n = 1305)	670 (51.3)	23 (28.4)	7 (14.6)	1 (20.0)	79 (32.1)	44 (65.7)	0 (00.0)	28 (51.9)	13 (6.3)	121 (80.1)	159 (100.0)	195 (96.1)
Pelvic floor exercises performed (Urinary fistula only; n = 1308)	1118 (85.5)	62 (73.8)	12 (25.5)	5 (100.0)	226 (91.9)	48 (72.7)	0 (00.0)	53 (96.4)	202 (97.1)	150 (100.0)	158 (98.8)	202 (100.0)
Prophylactic antibiotics provided (n = 1330)	1150 (86.5)	77 (93.9)	5 (10.0)	2 (40.0)	251 (100.0)	66 (97.1)	87 (100.0)	10 (17.86)	210 (100.0)	153 (100.0)	165 (100.0)	124 (61.1)

*BG* = Bangladesh, *GU* = Guinea, *NI* = Niger, *NG* = Nigeria, *UG* = Uganda.

*N/A* = Not applicable.

For women with urinary fistula only, the vaginal approach accounted for 95.1% (1215/1277) of repairs; the abdominal approach, for 4.1% (52/1277); and the combined, for 0.8% (10/1277). Women with RVF had the vaginal approach 95.5% (21/22) of the time; one case (4.5%) used the combined approach. All repairs of concurrent urinary fistula and RVF used the vaginal route.

There is some subjectivity to the indications for surgical route, varying by surgeon’s training, experience, and tradition in obstetrics/gynecology or urogynecology. Nevertheless, the crucial consideration should be ease of access to the tissues for repair. SIU-ICUD proposes vaginal route under ‘local’ anesthesia (understood to be ‘spinal’, rather than local infiltration) as the standard for most repairs [6]. The abdominal route may be needed for high-location fistulae where vaginal access is problematic such as for ureteric, some vesico-uterine and intracervical fistulae or when concomitant procedures like ureteral reimplantation, demand it.

Reimplantation of ureters was performed for 34 of 1319 women (2.6%) with a urinary fistula. Reimplantations are usually easier by abdominal rather than vaginal route, especially for high-location fistulae but over one third of the reimplantations (12/34, 35.3%) in this study were transvaginal. Reasons for this are unclear. The finding of ureteric openings within the margins of circumferential or VVF can often be managed by incorporating them into the repair during closure of the bladder defect by vaginal route, rather than reimplanting them separately.

Route of repair might influence fistula repair outcomes. Frajzyngier et al. in a different analysis of the data set in this study found that the vaginal route was associated with a higher risk of failed closure; stratified analyses found an elevated risk among women repaired vaginally who met indications for the abdominal route, but the small number of women undergoing abdominal repair limited the analysis [12]. In a retrospective study, Chigbu et al. reported successful closure in 84.3% of women repaired abdominally compared to 77.8% of those repaired vaginally, but the difference was not statistically significant [13]. In all cases, failure in the vaginal group occurred when the surgeon had difficulty accessing the repair site; all were subsequently repaired abdominally.

#### Intraoperative use of ureteric catheters

Intraoperative ureteric catheters were used for 9.4% (124/1313) of the urinary fistula repairs, presumably to locate and secure the ureters since most were removed before completion of surgery. Waaldijk [10] and Hancock [11] recommend intraoperative identification of the ureters by ureteric catheterization when proximity to the surgical field increases risk of ureteric injury. A ureteric catheter can also be used longer-term as a splint to facilitate healing of an injured ureter.

#### Relaxing incisions

Relaxing incisions were performed in 1.3% (16/1261) of repairs (Table 1). The low number might reflect surgeons’ attitude of the perceived risk versus benefits from such procedures and also the low proportions of severe vaginal scarring reported (in cases of severe scarring and stenosis relaxing incisions may be necessary to improve exposure of the operative field). Kirschner and colleagues reported that relaxing incisions were associated with twice the odds of incontinence at discharge (OR 1.91, 95%, CI 1.25–3.11) [14] but confounding by indications for relaxing incision cannot be ruled out.

Anecdotally, some surgeons use the term ‘relaxing incisions’ to include episiotomies. We did not specifically ask about episiotomy. The FIGO training manual includes episiotomy in its basic surgical steps, but clarifies that it may not be necessary for simple fistula if use of speculum and positioning on the table gives adequate access [5]. Waaldijk advocates for improving access when needed by performing unilateral, bilateral, or median episiotomy [10]. SIU-ICUD advises that, in addition to episiotomy, incision be performed if needed for any vaginal bands or strictures found, in order to improve exposure of the surgical field [6].

#### Suturing techniques and use of flaps and grafts

The bladder was closed in a single layer for nearly two-thirds of women with urinary fistula (820/1271, 64.5%) and in double layers for 33.8% (428/1265), with variation across sites (Table 1). Nardos et al. reported that single-layer bladder closure performed as well as double-layer closure [15]. Hancock cautioned against use of a second layer of bladder suture when suturing to the proximal urethra, as there is a risk of cutting through or shortening the urethra, unless there is adequate tissue [11]. The vagina was repaired in a single layer for most women with urinary fistula (1076/1269, 84.8%), with use of single-layer suturing common at all but one site, NI2 (Table 1). Double-layer vaginal wall closure was uncommon (14/1261, 1.1%).

For RVF, the rectum was repaired in a single layer for 50.8% (32/63) of women and in double layers for 34.4% (21/61). Vaginal closure was in a single layer for 70.0% (42/60) of women with RVF and in double layers for 5.0% (3/60).

Intraoperative grafts or flaps (the terms were used to refer to the same procedures) were rarely used. In women with urinary fistula, surgeons recorded use of the Martius graft in 3.4% (43/1263) of cases and an omental flap in 0.2% (3/1263) (Table 1). For RVF with or without urinary fistula, the Martius graft was used in only one of 60 cases. Theoretically, flaps and grafts protect the fistula closure and help achieve continence by improving support and blood supply to the repair site. Once commonly used, they are becoming less common [11], and many surgeons now use them only in special circumstances, such as when the urethra has to be reconstructed, when tissues are particularly poor quality, or when there have been multiple previous attempts at repair [6]. The SIU –ICUD notes that, when the Martius graft is used as a sling, it does not help to achieve continence and could result in significant surgical trauma [6]. Browning found that a significantly higher proportion of women with a Martius flap experience residual incontinence; however, stratified analyses demonstrated that fistula repaired with Martius flap may have been more difficult. Residual confounding by indication cannot be ruled out [16].

#### Ancillary procedures to avoid post-repair incontinence

A total of 75 out of 1334 women (5.6%) received ancillary procedures intraoperatively to reduce the likelihood of post-repair incontinence. Some women received more than one procedure, for a total of 85 procedures. Most frequent was urethral elongation/reconstruction (45/75 women, 60.0%), a finding similar to Arrowsmith et al.’s report [7]. The second most frequent was retropubic cystourethropexy (27/75 women, 36.0%), primarily paravaginal colposuspension (n = 23). Other ancillary procedures were rare; pubovaginal sling (n = 6), Browning sling (n = 4), plication sutures (n = 2), and buttress sutures (n = 1).

Many surgeons believe that the risk of postoperative incontinence can be reduced by technical interventions done as part of the fistula repair itself and by use of ancillary intraoperative procedures like those we observed [7], although reported effectiveness of different procedures to decrease post-repair incontinence varies widely, and spontaneous improvement occasionally happens over time [6]. Nonetheless, SIU-ICUD recommends that when risk factors for residual incontinence are identified, that they be managed at the time of fistula closure with measures such as urethral lengthening, slings and suspensions [6]. These and other anti-incontinence procedures can also be performed at a later date if risk of incontinence is not apparent at the time of the repair or there are other reasons to delay an intraoperative intervention.

#### Duration of operation

Median duration of operation was nearly 1 hour (54 minutes, IQR 35–90 minutes) from incision to last suture, with a range of 15 minutes to 5 hours. This is similar to the wide range in duration reported by at least one other author who also noted that, for inexperienced surgeons, faulty technique or selection of cases that are too complex for their skill level could make for unduly long operations [11]. For the woman’s safety and because of implications for type of anesthesia needed, it is imperative to match case complexity to the provider skill level so that the operation does not carry on for longer than necessary.

#### Assessing fistula closure outcome at the end of operation

To assess fistula closure at the end of surgery, a dye test was done for 65.6% (850/1295) of women with urinary fistulae (including combined fistulae). The closure rate among all women at the end of urinary fistula repair surgery was 95.8% (1245/1299) (see Table 2). Some had a dye test and some (approximately one-third of cases) did not; notably, dye test is considered a standard step in FIGO’s training manual, to confirm closure, integrity of the repair and also that there is no additional fistula that has been overlooked [5].

**Table 2:** Fistula closure and residual incontinence outcomes at discharge and 3 month follow-up, by site

	**Site N (%)**											
	**Total (n = 1354)**	**BG 1 (n = 85)**	**BG 2 (n = 50)**	**BG 3 (n = 5)**	**GU (n = 251)**	**NI 1 (n = 74)**	**NI 2 (n = 97)**	**NG 1 (n = 59)**	**NG 2 (n = 210)**	**NG 3 (n = 153)**	**UG 1 (n = 165)**	**UG 2 (n = 205)**
Urinary fistula closure at end of repair surgery (n = 1299)	1245 (95.8)	73 (88.0)	45 (93.8)	5 (100.0)	231 (93.9)	63 (95.5)	89 (100)	50 (94.3)	195 (95.1)	141 (94.6)	155 (99.4)	198 (99.5)
Urinary fistula closure at discharge (n = 1307)	1104 (84.5)	62 (75.6)	34 (70.8)	5 (100.0)	204 (82.9)	40 (59.7)	72 (86.8)	39 (72.2)	189 (90.9)	115 (76.5)	141 (88.1)	203 (100.0)
Urinary fistula closure at 3 month follow-up (n = 1274)	1039 (81.6)	52 (73.2)	34 (70.8)	3 (60.0)	185 (75.2)	40 (59.7)	45 (66.2)	37 (69.8)	190 (91.4)	117 (80.1)	138 (86.8)	198 (97.5)
Residual incontinence at discharge among women with urinary fistula (n = 1084)	219 (20.2)	19 (32.8)	9 (27.3)	2 (66.7)	28 (13.7)	3 (7.9)	17 (24.3)	12 (31.6)	21 (11.2)	35 (31.0)	26 (18.6)	47 23.62
Residual incontinence at 3 months follow-up among women with urinary fistula (n = 1015)	192 (18.9)	9 (19.2)	16 (47.1)	3 (100.0)	23 (12.5)	4 (10.5)	18 (40.9)	8 (21.6)	18 (9.8)	34 (29.1)	15 (10.9)	44 (23.3)
RVF Closure at discharge (n = 47)	44 (93.6)	1 (100.0)	2 (100.0)	N/A	9 (90.0)	2 (100.0)	4 (100.0)	3 (100.0)	5 (71.4)	6 (100.0)	5 (100.0)	7 (100.0)
RVF Closure at 3 month follow-up (n = 43)	36 (83.7)	1 (100.0)	1 (50.0)	N/A	8 (80.0)	3 (100.0)	2 (100.0)	3 (100.0)	3 (75.0)	3 (50.0)	5 (100.0)	7 (100.0)

*BG* = Bangladesh, *GU* = Guinea, *NI* = Niger, *NG* = Nigeria, *UG* = Uganda.

*N/A* = not applicable.

### Postoperative procedures and practices

#### Vaginal packs

Vaginal packs were used for almost all women (1246/1328, 93.8%); in most cases they were left in place for 1 (826/1246, 66.3%) or 2 (396/1246, 31.8%) days. The pack was left in for 3 or more days in 1.9% (24/1246) of women. Opinions regarding the benefit of vaginal packs are conflicting. While they might encourage the various layers of the repair to adhere, minimizing dead space and hematoma formation, they could have a drawback in preventing early recognition of deep bleeding [11]. The FIGO training manual states that vaginal packs are optional and, if used, should be removed in 24–72 hours. The surgeons in our study were for the most part in line with this recommendation for duration of use. If left in continuously and too long there is grave risk for local and systemic sepsis. While simple cases may not need vaginal packs, Hancock has suggested an all-or-none policy (i.e. site level decision to use packs for all of the cases or none of the cases) so as to simplify nursing care and avoid confusion which could result in a retained vaginal pack and disastrous consequences [11]. Indeed, in our study, five sites used vaginal packs for all cases as a matter of course.

#### Strict postoperative bed rest

The median duration of strict bed rest following repair was 1 day (IQR 1–3 days). While the range was 0–24 days, most women were on strict bed rest for 2 (530/1331, 39.8%) or 3 days (402/1331, 30.2%). More than 5 days was reported for approximately 10% (141/1331, 10.6%) of the women. Median duration of strict bed rest varied among sites (Table 1). Duration of strict bed rest varied by route of repair: 2–5 days for most women with abdominal repair (40/51, 78.4%), compared to 1–3 days for most women after vaginal repair (893/1268, 70.4%). While lying flat on the back for 24 hours has been found to reduce the risk of post-spinal anesthesia headache, there is no evidence that prolonged bed rest is beneficial; it could, in fact, increase the risk of deep vein thrombosis, pulmonary stasis, etc. The FIGO manual recommends mobilization as soon as possible [5].

#### Postoperative bladder catheterization

Nearly all women who had a urinary fistula repair had an indwelling bladder catheter inserted at the end of surgery (1310/1313, 99.8%). The balloon was filled with a median of 5 cc (IQR 5–10 cc) air or fluid.

Just over two-thirds (899/1310, 68.6%) of women had open urinary bladder drainage after repair of urinary fistula; by contrast, in the Arrowsmith et al. survey, one-third of respondents reported use of open drainage [7]. There was wide variation among sites (Table 1). At three sites, not a single woman was managed with open drainage; at three other sites, all women were managed with open drainage.

The decision of whether to use open or closed drainage may align more with site resources than with fistula characteristics. Closed drainage can be more expensive in terms of supplies and requires quality urine bags, which may not be available. Additionally there must be adequate nursing staff to allow for regular monitoring of urine bags, which may overfill and result in the catheter balloon exerting pressure on the repair with resultant damage. Theoretically, chances of ascending infection might be less than for open drainage, but closed drainage could keep a woman in bed longer, which can increase the risk of thromboembolism.

For 1294 women who had urinary fistula repair, the median duration of postoperative urethral catheterization was 21 days (IQR 14–27). Median duration varied by site (Table 1), ranging from 14 to 29 days. Most women (894/1294, 69.1%) had their catheter in place for more than 14 days after repair surgery. Duration of catheterization correlated with perceived difficulty of the repair as subjectively determined during the EUA, with longer durations following repair of more complex fistula. Among women whose fistula was classified as simple, 56.8% (208/366) were catheterized for longer than 14 days, compared to 71.9% (400/556) for intermediate fistula and 77.7% (283/364) for complex fistula. These findings mirror those of others who also found that patients with more damage (e.g., more vaginal scarring, a larger fistula, or greater urethral damage) had their catheter in place for a longer period [7,17]. Duration of catheterization is a key determinant for length of hospital stay, which impacts treatment costs and efficient use of bed space, but there is little published evidence on which to base decisions about duration of catheterization.

#### Postoperative bladder training before discharge

Approximately half of the women who had a urinary fistula repair (670/1305, 51.3%) received bladder training before discharge. Reported use of bladder training varied among sites (Table 1). Prolonged intervals between micturition was the most frequently reported approach (382/1300, 29.4%), followed by intermittent catheter clamping (270/1300, 20.8%). During discharge interviews, most women (1118/1308, 85.5%) with a urinary fistula reported doing pelvic floor exercises during their hospital stay, with some variation among sites (Table 1).

Recovery of bladder and sphincter function can take variable amounts of time and there is limited empirical evidence on whether or not bladder training or pelvic floor therapy improves these functions or shortens recovery time. Intermittent clamping in particular has potential for serious consequences, including repair breakdown from severe backpressure, especially when nursing staffing levels are low, supervision is minimal, and the clamp is left on too long [6]. The fact that intermittent clamping was reportedly used in 20% of the participants in our study requires further attention because it could potentially result in repair breakdown from back pressure if the clamp is inadvertently left on too long.

#### Determining outcome at discharge

Status of the urinary fistula at the time of catheter removal or discharge was determined by a dye test for 64.8% (700/1081) of women. Outcome in the remaining women was determined based on pelvic exam findings and/or the presence/absence of continuous uncontrolled urine leakage. Overall, 84.5% (1104/1307) of urinary fistulae were reportedly closed at discharge, with variation among the sites (Table 2). These rates are in line with what was reported by SIU-ICUD [6].

Hancock advised performing a dye test just before catheter removal to determine closure and to note any urethral leakage [11], while the FIGO training Manual recommends a pre-discharge dye test be ‘considered’ [5]. About one-fourth of surgeons surveyed by Arrowsmith et al. [7] reported they routinely performed dye test before catheter removal.

The RVF (with or without a concurrent urinary fistula) closure rate at discharge was 93.6% (44/47), but varied by site (Table 2).

#### Pre-, intra-, and postoperative drinking regimens

A preoperative drinking regimen was prescribed for 80.2% of women with a urinary fistula (1054/1314); numbers varied by site, from none of the women at two sites to all women at two other sites (Table 1). The median volume of water prescribed was 5 liters (IQR 4–8), and in line with FIGO Global Training Manual recommendation of 3–5 liters [5].

Left to their own devices, women are likely to come to theater dehydrated with urine highly concentrated with irritant phosphates and nitrates because they are trying to reduce wetness [11]. Such women are more likely to become hypotensive under spinal anesthesia. Also, the surgeon may have difficulty identifying ureteric orifices, and urine output will be poor, predisposing the woman to catheter blockage or requiring more postoperative IV fluids. In addition, pre- and/or postoperative drinking regimens may reduce perioperative infections and improve bladder capacity, and they can be used to evaluate compliance with the perioperative regimen. Other surgeons do not prescribe drinking regimens and have not reported increased problems with perioperative infections or catheter blockage [6]. In theory, women face a risk of hyponatremia if they drink too much water, a complication that, although rare, is potentially fatal [11]. One challenge faced by low-resource programs may be difficulty obtaining large volumes of safe drinking water; buying bottled water can be expensive and logistically difficult.

Intraoperative IV fluids were administered during the majority of repairs (1270/1340, 94.8%). Part of the reason could be to forestall hypotension, an occasional complication of spinal anesthesia or to have an intravenous line ready in case it is needed urgently. Transabdominal surgeries could result in temporary intestinal and peristaltic dysfunction; each woman who underwent an abdominal or combined vaginal/abdominal approach received IV fluids during surgery. Waaldijk reports only providing fluids if blood pressure falls below 80 mm Hg [10]). The FIGO manual, while specific for pre- and post- operative fluids needed (3–5 liters per day) is less prescriptive for the intra-operative phase, merely calling for ‘adequate fluid management’ [5].

Postoperative drinking regimen was prescribed for 88.0% (1148/1304) of women who had a urinary fistula repaired, with a median fluid volume of 5 liters (IQR 3–8) per day. The rationale is similar to the one for preoperative drinking regimens. Although there was some variability among sites, seven of the 11 sites prescribed a drinking regimen to all of the women (Table 1).

#### Antibiotic use in Pre-, Intra-, and postoperative periods

Antibiotic use was relatively common before, during and after repair, primarily for prophylactic purposes. Just over 40% of the women received prophylactic antibiotics prior to surgery (552/1340, 41.2%), nearly two-thirds received them intraoperatively (843/1340, 62.9%), and 1150 of 1330 women (86.5%) received postoperative prophylactic antibiotics. Although prophylactic antibiotic use varied widely among the sites at all three time points, it appeared to be routine at most facilities in the intra- and postoperative periods (Table 1). Antibiotics for therapeutic purposes were rare.

Prophylactic antibiotics use varied by type of fistula primarily when administered preoperatively; fewer (23.8%, 10/42) women with combined fistula received preoperative prophylactic antibiotics than women with urinary fistula (41.7%, 532/1276) or women with RVF (45.5%, 10/22). Intraoperative prophylactic antibiotic use did not vary by type of fistula. All women with an RVF (22/22, 100.0%) and most women with a urinary (1091/1267, 86.1%) or combined fistula (37/41, 90.2%) received prophylactic antibiotics during the postoperative period.

Arrowsmith et al’s survey similarly found that the majority of respondents gave prophylactic antibiotics; however, respondents revealed that prophylaxis sometimes ended up being empirical therapy rather than prophylaxis. More than half the respondents reported using antibiotics for every surgical case; but there was little agreement about what type of antibiotic and regimen to use [7].

It would be beneficial to standardize definitions and guidelines for prophylactic antibiotic use in fistula repair services and training programs so as to reduce both costs and potential development of antibiotic resistance [6,7]. Unfortunately the evidence base to inform appropriate antibiotic use is sparse and inconclusive, with one report finding no benefit of single dose prophylaxis over no antibiotics [18] and another showing marginal benefit of a single dose given at the start of surgery compared to antibiotic use for the 7 days after surgery [19]. It may be possible, however, to develop appropriate recommendations for fistula repair surgery based on data and guidance available for other types of pelvic surgery [20,21]. The FIGO Manual recommends prophylactic antibiotics, without details on timing; but urging considerations such as sensitivity patterns and awareness of what other antibiotics the woman might be already on [5]. SIU-ICUD notes, that while there are no good data in the literature on antibiotic prophylaxis for fistula repair, following international guidelines for surgery generally, one single prophylactic dose of a broad-spectrum antibiotic at induction of anesthesia before surgery could be recommended [6].

#### Fertility-related counseling and provision of family planning

Most women reported receiving counseling on fertility potential, resumption of sexual activity, planning for the next delivery, and family planning use (results related to counseling and services on other topics are reported elsewhere [9]). While overall levels of counseling were reported to be quite high, findings varied with site and topic (Table 3). Counseling could be beneficial in avoiding and managing cases of early repair breakdown [11] as well as longer- term recurrence of successfully repaired fistula [6].

**Table 3:** Fertility-related counseling during hospitalization and family planning method provision among women having fistula repair surgery

	**Site N (%)**											
**Characteristic**	**Total (n = 1354)**	**BG 1 (n = 85)**	**BG 2 (n = 50)**	**BG 3 (n = 5)**	**GU (n = 251)**	**NI 1 (n = 74)**	**NI 2 (n = 97)**	**NG 1 (n = 59)**	**NG 2 (n = 210)**	**NG 3 (n = 153)**	**UG 1 (n = 165)**	**UG 2 (n = 205)**
Counseled about^1^												
Fertility potential (n = 1322)	1065 (80.6)	78 (92.9)	27 (57.4)	4 (80.0)	238 (95.2)	67 (98.5)	83 (100.0)	56 (98.2)	174 (83.3)	149 (98.7)	165 (100.0)	24 (11.8)
Resuming sexual intercourse (n = 1335)	1274 (95.4)	80 (94.1)	45 (91.8)	4 (80.0)	250 (99.6)	69 (100.0)	89 (100.0)	57 (100.0)	160 (76.6)	151 (99.3)	165 (100.0)	204 (100.0)
Planning for next delivery (n = 1319)	1226 (92.9)	71 (85.5)	28 (59.6)	3 (75.0)	237 (94.8)	68 (98.6)	82 (98.8)	56 (98.2)	165 (79.3)	150 (99.3)	165 (100.0)	201 (99.5)
Family planning method use (n = 1325)	1205 (90.9)	67 (79.8)	26 (55.3)	3 (75.0)	237 (94.8)	69 (100)	86 (100.0)	57 (100.0)	174 (83.3)	135 (88.8)	164 (99.4)	187 (92.6)
Received family planning method at discharge (n = 1328)	785 (59.1)	18 (22.0)	1 (2.0)	0 (0.0)	182 (72.5)	55 (79.7)	83 (93.3)	3 (5.6)	173 (82.8)	61 (40.4)	27 (16.4)	182 (89.2)
Injectable contraceptives	343 (43.7)	0 (0.0)	0 (0.0)	N/A	74 (40.7)	32 (58.2)	54 (65.1)	1 (33.3)	152 (87.9)	17 (27.9)	13 (48.1)	0 (0.0)
Oral contraceptives	188 (23.9)	13 (72.2)	1 (100.0)	N/A	106 (58.2)	13 (23.6)	29 (34.9)	0 (0.0)	2 (1.2)	19 (31.1)	5 (18.5)	0 (0.0)
Natural family planning/calendar method	183 (23.3)	0 (0.0)	0 (0.0)	N/A	0 (0.0)	0 (0.0)	0 (0.0)	0 (0.0)	1 (0.6)	0 (0.0)	0 (0.0)	182 (23.3)
Tubal ligation	12 (1.5)	0 (0.0)	0 (0.0)	N/A	0 (0.0)	5 (9.1)	0 (0.0)	0 (0.0)	0 (0.0)	1 (1.6)	6 (22.2)	0 (0.0)
Contraceptive implant	3 (0.4)	0 (0.0)	0 (0.0)	N/A	0 (0.0)	3 (5.5)	0 (0.0)	0 (0.0)	0 (0.0)	0 (0.0)	0 (0.0)	0 (0.4)
Intrauterine device	1 (0.1)	0 (0.0)	0 (0.0)	N/A	0 (0.0)	0 (0.0)	0 (0.0)	0 (0.0)	0 (0.0)	0 (0.0)	1 (3.7)	0 (0.0)
Method not specified	55 (7.0)	5 (27.8)	0 (0.0)	N/A	2 (1.1)	2 (3.6)	0 (0.0)	2 (66.7)	18 (10.4)	24 (39.3)	2 (7.4)	0 (0.0)

*BG* = Bangladesh, *GU* = Guinea, *NI* = Niger, *NG* = Nigeria, *UG* = Uganda.

*N/A* = Not applicable.

^1^ Multiple responses allowed; percentages may exceed 100%.

Most (1205/1325, 90.0%) women reported being counseled about family planning during their hospital stay, with some differences between sites (Table 3). Over half (785/1328, 59.1%) of women said they received a family planning method before discharge. Among women who said they received a method, the most widely dispensed method was an injectable (343/785, 43.7%), followed by oral contraceptives (188/785, 23.9%) and counseling for natural family planning (183/785, 23.3%). Few women (16/785, 2.0%) reported receiving long-acting or permanent methods such as tubal ligation, a contraceptive implant, or an intrauterine device. It is important that all women be offered holistic counseling regarding fertility potential and ways to achieve their fertility intentions and referral if needed for infertility and sub-fertility management, which are problematic for many of the women following fistula repair [6].

### Procedures for diagnosis and management of early breakdown and residual incontinence

Fistula closure at 3 months was determined by pelvic examination, with a dye test in women who reported urine leakage. When no pelvic examination or dye test was conducted (186/1300, 14.6%), fistula closure was determined by the provider’s response to the question “Does the client have continuous and uncontrolled leakage of urine?” Overall, urinary fistula closure at 3 months was 81.6% (1039/1274), although rates varied widely by site (Table 2).

Interestingly, two sites reported a small improvement in closure rates at three months (i.e., women discharged wet but coming back dry). Conversely, 41 women who were dry at discharge came back wet at 3 months (i.e., they experienced an early post-discharge breakdown). If such a case is suspected, the surgeon should question the woman carefully to try to determine the cause (e.g., over-distension of the bladder, early intercourse, infection) because, if women come back at once, small breakdowns can often be repaired or cured by placing an indwelling bladder catheter [11]. Counseling is therefore important to make sure women know that they should come back as soon as possible if they start leaking again.

Post-repair residual incontinence (i.e., some incontinence remaining after fistula closure) has been reported as a significant problem; some view it as a failure of the intervention since the woman is not restored to her pre-fistula continence state [7]. While most women with repaired urinary fistula (823/1041, 79.1%) were closed and dry at 3 months, nearly 20% (192/1015, 18.9%) had residual incontinence at 3 months post-repair, with rates varying widely among sites (Table 2). The most frequent type of incontinence was reportedly stress (169/1053, 16.0%), but formal urodynamic studies were not readily available at the study sites and diagnosis was primarily based on history and physical exam findings. Results of the survey by Arrowsmith et al. [7] also found that most surgeons reported they diagnosed post-repair urinary incontinence using a combination of history, physical examination, and a negative dye test.

## Conclusions

This was one of the largest prospective multi-country studies of fistula repair services, covering a wide geographic area and a variety of economic and organizational conditions. The analyses presented here demonstrate wide variability in many perioperative procedures and practices, a finding that is not surprising given the lack of guidelines until recently and a dearth of empirical research evaluating the effectiveness of many of these procedures. A recent systematic review by Frajzyngier et al. [22] found that that only a small number of studies to date have empirically examined the effectiveness of various peri-operative procedures on repair outcomes; most were limited by small sample sizes and did not use rigorous epidemiologic research methods; confounding could not be excluded as an explanation for the results of many of these studies. Similarly, SIU-ICUD notes that the level of evidence for much of the research remains weak as many of the reports are based on personal case series [6].

The analyses presented here do not allow us to propose definitive changes, but the wide range in procedures and practices indicates that there is need for standardization. While the extent to which the recent guidelines have been rolled out and implemented in programs is still in question, their full utility will also continue to be unclear, given the continued lack of compelling evidence for optimal practices. Is it even reasonable to expect standardization of such practices without support from empirical studies? Further research is called for, especially on procedures that have particular implications for safety and cost effectiveness, including:

• The role of antibiotics in prophylaxis and the incidence of multi-drug resistant organisms; related practices have serious implications for financial and morbidity costs.

• The usefulness and safety of pre and postoperative fluid regimens.

• Best ancillary procedures for prevention of residual incontinence and the role of simple bed-side as well as formal urodynamic studies in its diagnosis and treatment.

• Catheter management for fistulae of different complexity levels; duration of catheterization, type of urinary drainage and need for bed rest or for in-patient management.

• The role of different techniques for bladder training and for pelvic floor rehabilitation in the treatment of postoperative pelvic floor dysfunction.

Finally, operational research is needed on counseling, its role in treatment outcomes and how to best support women’s longer term reproductive intentions, including pregnancy or birth spacing.

## Abbreviations

FIGO: International Federation of Gynaecology and Obstetrics; ICUD: International Consultation on Urological Disease; RVF: Rectovaginal Fistula; SIU: Societe Internationale d’Urologie; USAID: United States Agency for International Development; VVF: Vesicovaginal fistula.

## Competing interests

The authors declare that there are no competing interests.

## Authors’ contributions

JKR, MAB, and VF made principal contributions to the research design, analyses, and drafting of the paper. MBA, FA, THB, AB, DD, SOG, SI, MI, ML, FM, DCP and AW made contributions to study implementation and critical review of the paper. All authors read and approved the final manuscript.

## Pre-publication history

The pre-publication history for this paper can be accessed here:

http://www.biomedcentral.com/1471-2393/14/220/prepub
